# Supplementary material for: Factors leading to the late diagnosis and poor outcomes of breast cancer in Matabeleland South and the Bulawayo Metropolitan Provinces in Zimbabwe
Source: PLoS One. 2023 Nov 3;18(11):e0292169. doi: 10.1371/journal.pone.0292169 (PMC10624273; doi:10.1371/journal.pone.0292169)
Supplement: S1 File — (DOCX) [file pone.0292169.s001.docx]

**Table (i):** **Number of respondents per district**

| **District** | **Total** | **(%)** |
| --- | --- | --- |
| Beitbridge | 103 | 9.3 |
| Bulilima | 39 | 3.5 |
| Gwanda | 108 | 9.8 |
| Insiza | 110 | 9.9 |
| Mangwe | 61 | 5.5 |
| Matobo | 96 | 8.7 |
| Umzingwane | 94 | 8.5 |
| Bulawayo Metropolitan | 477 | 43.1 |
| **Total** | **1088** | **98.3** |

**Table (ii):** **Gender versus education level.**

| **Gender** | **Education frequencies** | | | | | | **Total** |
| --- | --- | --- | --- | --- | --- | --- | --- |
|  | **Degree** | **Diploma** | **Advanced level** | **Ordinary level** | **Grade 7** | **No formal education** |  |
| Male | 25 | 36 | 19 | 75 | 23 | 5 | **183** |
| Female | 86 | 140 | 75 | 427 | 144 | 35 | **907** |
| **Total** | **111** | **176** | **94** | **502** | **167** | **40** | **1090** |
| **Total(%)** | **10.0** | **15.9** | **8.5** | **45.3** | **15.1** | **3.6** | **98.4** |

| **Table (iii): Gender and Knowledge of BC** | | | | | | |
| --- | --- | --- | --- | --- | --- | --- |
|  |  |  | Know | | | Total |
|  |  |  | Yes | No | Don't know |  |
| Gender | Male | Count | 103 | 62 | 18 | 183 |
|  |  | % within Gender | 56.3% | 33.9% | 9.8% | 100.0% |
|  |  | % within Know | 14.9% | 19.3% | 25.4% | 16.9% |
|  |  | % of Total | 9.5% | 5.7% | 1.7% | 16.9% |
|  | Female | Count | 587 | 260 | 53 | 900 |
|  |  | % within Gender | 65.2% | 28.9% | 5.9% | 100.0% |
|  |  | % within Know | 85.1% | 80.7% | 74.6% | 83.1% |
|  |  | % of Total | 54.2% | 24.0% | 4.9% | 83.1% |
| Total | | Count | 690 | 322 | 71 | 1083 |
|  |  | % within Gender | 63.7% | 29.7% | 6.6% | 100.0% |
|  |  | % within Know | 100.0% | 100.0% | 100.0% | 100.0% |
|  |  | % of Total | 63.7% | 29.7% | 6.6% | 100.0% |

**Table (iv):** **Knowledge of breast lumps by age group.**

| **Age group** | | **Knowledge of breast lumps** | | | |
| --- | --- | --- | --- | --- | --- |
|  |  | **Yes** | **No** | **Don’t know** | **Total** |
| Age group  < 30 years | Count within age | 201 | 114 | 37 | 352 |
|  | Age group (%) | 57.1 | 32.4 | 10.5 | 100.0 |
|  | Know anything about breast lumps? (%) | 29.3 | 36.0 | 52.9 | 32.8 |
|  | Total (%) | 18.8 | 10.6 | 3.5 | 32.8 |
| 30-39 years | Count within age | 235 | 95 | 17 | 347 |
|  | Age group (%) | 67.7 | 27.4 | 4.9 | 100.0 |
|  | Know anything about breast lumps? (%) | 34.3 | 30.0 | 24.3 | 32.4 |
|  | Total (%) | 21.9 | 8.9 | 1.6 | 32.4 |
| 40-49 years | Count within age | 149 | 62 | 13 | 224 |
|  | Age group (%) | 66.5 | 27.7 | 5.8 | 100.0 |
|  | Know anything about breast lumps? (%) | 21.8 | 19.8 | 18.6 | 20.9 |
|  | Total (%) | 13.9 | 5.8 | 1.2 | 20.9 |
| > 50 years | Count within age | 100 | 46 | 3 | 149 |
|  | Age group (%) | 67.1 | 30.9 | 2.0 | 100.0 |
|  | Know anything about breast lumps? (%) | 14.6 | 14.5 | 4.3 | 13.9 |
|  | Total (%) | 9.3 | 4.3 | 0.3 | 13.9 |
| **Overall** | **Count** | **685** | **317** | **70** | **1072** |
|  | **Total (%)** | **63.6** | **29.4** | **6.5** | **99.5** |
|  |  |  |  |  |  |

**Table (v):** **Knowledge of breast lumps by district.**

| **District** | | **What is breast cancer?** | | | |
| --- | --- | --- | --- | --- | --- |
|  |  | **Growth** | **Infection** | **Other** | **Total** |
| Beitbridge | Count | 64 | 23 | 5 | 92 |
|  | Total (%) | 6.4 | 2.3 | 0.5 | 9.1 |
| Bulilima | Count | 18 | 14 | 3 | 35 |
|  | Total (%) | 1.8 | 1.4 | 0.3 | 3.5 |
| Gwanda | Count | 84 | 20 | 1 | 105 |
|  | Total (%) | 8.3 | 2.0 | 0.1 | 10.4 |
| Insiza | Count | 58 | 46 | 0 | 104 |
|  | Total (%) | 5.8 | 4.6 | 0 | 10.3 |
| Mangwe | Count | 38 | 19 | 2 | 59 |
|  | Total (%) | 3.8 | 1.9 | 0.2 | 5.9 |
| Matobo | Count | 45 | 37 | 7 | 89 |
|  | Total (%) | 4.5 | 3.7 | 0.7 | 8.8 |
| Umzingwane | Count | 43 | 33 | 0 | 76 |
|  | Total (%) | 4.3 | 3.3 | 0 | 7.5 |
| Bulawayo Metropolitan | Count | 310 | 108 | 29 | 447 |
|  | Total (%) | 30.8 | 10.7 | 2.9 | 44.4 |
| **Total** | **Count** | **660** | **300** | **47** | **1007** |
|  | **Total (%)** | **65.5** | **29.8** | **4.7** | **100.0** |

**Table (vi):** **Other definitions of breast cancer by respondents.**

| **What is breast cancer? If other, specify.** | **Number** | **(%)** |
| --- | --- | --- |
| Black magic | 1 | 0.1 |
| Don’t know | 5 | 0.5 |
| Evil spirits | 1 | 0.1 |
| Imvukuzane (a mole) | 8 | 0.7 |
| It’s a disease | 1 | 0.1 |
| Mbeva (a mouse) | 3 | 0.3 |
| Mice | 1 | 0.1 |
| No idea | 1 | 0.1 |
| Not sure | 3 | 0.3 |
| Spiritual disease | 1 | 0.1 |
| Tumour | 2 | 0.2 |
| Witchcraft | 6 | 0.5 |

**Table (vii):** **Reasons for delays.**

| **Delays** | **Number** | **(%)** |
| --- | --- | --- |
| Inadequate medicine | 49 | 18.7 |
| Inadequate equipment | 36 | 13.7 |
| Poor access to clinic/ hospital | 34 | 13.0 |
| Traditional/ cultural beliefs | 24 | 9.2 |
| Religious beliefs | 17 | 6.5 |
| Poor knowledge of disease | 58 | 22.1 |
| Economic challenges | 44 | 16.8 |
| **Total** | **262** | **100.0** |

**Table (viii): Mode of transport to hospitals or clinics.**

| **Mode of transport** | **Number** | **(%)** |
| --- | --- | --- |
| On foot | 259 | 23.4 |
| Scotch cart | 15 | 1.4 |
| Bicycle | 18 | 1.6 |
| Bus or car | 132 | 11.9 |
| Own transport | 29 | 2.6 |
| No response given | 654 | 59.1 |
| **Total** | **1107** | **100.0** |

**Table (ix): Specialisation of health professionals by age group.**

| **Age group** | **Area of specialisation** | | | | | | | | **Total** |
| --- | --- | --- | --- | --- | --- | --- | --- | --- | --- |
|  | **Surgery** | **Radiography** | **Radiology** | **Pathology** | **Oncology** | **Nurse** | **Midwife** | **Other** |  |
| < 30 years | 0 | 2 | 1 | 0 | 0 | 53 | 1 | 6 | 63 |
| 30-39 years | 10 | 7 | 0 | 1 | 1 | 74 | 27 | 19 | 139 |
| 40-49 years | 1 | 3 | 0 | 2 | 0 | 21 | 22 | 6 | 55 |
| > 50 years | 1 | 0 | 1 | 0 | 0 | 10 | 13 | 12 | 37 |
| **Total** | **12** | **12** | **2** | **3** | **1** | **158** | **63** | **43** | **294** |

**Table (x): Work experience of health professionals by district.**

| **District** | **Experience in years** | | | | | **Total** |
| --- | --- | --- | --- | --- | --- | --- |
|  | **< 5** | **5-10** | **11-20** | **21-30** | **> 30** |  |
| Beitbridge | 7 | 7 | 8 | 1 | 0 | 23 |
| Bulilima | 1 | 0 | 1 | 0 | 0 | 2 |
| Gwanda | 6 | 8 | 5 | 1 | 0 | 20 |
| Insiza | 6 | 4 | 5 | 0 | 0 | 15 |
| Mangwe | 4 | 2 | 5 | 3 | 1 | 15 |
| Matobo | 4 | 3 | 10 | 0 | 0 | 17 |
| Umzingwane | 5 | 6 | 6 | 1 | 1 | 19 |
| Bulawayo Metropolitan | 61 | 38 | 51 | 17 | 18 | 185 |
| **Total** | **94** | **68** | **91** | **23** | **20** | **296** |

**Table (xi): Awareness of breast cancer incidence by age group.**

| **Age group** | **Awareness of breast cancer** | | | **Total** |
| --- | --- | --- | --- | --- |
|  | **Yes** | **No** | **Don’t know** |  |
| < 30 years | 42 | 15 | 8 | 65 |
| 30-39 years | 59 | 68 | 10 | 137 |
| 30-49 years | 31 | 19 | 3 | 53 |
| > 50 years | 27 | 8 | 2 | 37 |
| **Total** | **159** | **110** | **23** | **292** |

**Table (xii): Health professionals’ knowledge and support for the breast cancer project.**

| **District** | **Knowledge of the project** | | | **Support for the project** | | |
| --- | --- | --- | --- | --- | --- | --- |
|  | **Yes** | **No** | **Don’t know** | **Yes** | **No** | **Don’t know** |
| Beitbridge | 0 | 21 | 2 | 18 | 3 | 1 |
| Bulilima | 0 | 2 | 0 | 0 | 1 | 1 |
| Gwanda | 14 | 5 | 0 | 19 | 0 | 0 |
| Insiza | 4 | 9 | 2 | 14 | 1 | 0 |
| Mangwe | 7 | 8 | 0 | 15 | 0 | 0 |
| Matobo | 5 | 11 | 1 | 14 | 1 | 2 |
| Umzingwane | 3 | 13 | 3 | 12 | 4 | 3 |
| Bulawayo Metropolitan | 67 | 87 | 24 | 157 | 11 | 10 |
| **Total** | **100** | **156** | **32** | **249** | **21** | **17** |

**Table (xiii): Availability of histopathology laboratories.**

| **District** | **Sufficient laboratories** | | | **Total** |
| --- | --- | --- | --- | --- |
|  | **Yes** | **No** | **Don’t know** |  |
| Beitbridge | 1 | 10 | 11 | 22 |
| Bulilima | 0 | 1 | 1 | 2 |
| Gwanda | 1 | 0 | 19 | 20 |
| Insiza | 0 | 7 | 7 | 14 |
| Mangwe | 1 | 9 | 5 | 15 |
| Matobo | 0 | 8 | 9 | 17 |
| Umzingwane | 0 | 9 | 10 | 19 |
| Bulawayo Metropolitan | 22 | 93 | 70 | 185 |
| **Total** | **25** | **137** | **132** | **294** |
