# Supplementary material for: Factors leading to the late diagnosis and poor outcomes of breast cancer in Matabeleland South and the Bulawayo Metropolitan Provinces in Zimbabwe
Source: PLoS One. 2023 Nov 3;18(11):e0292169. doi: 10.1371/journal.pone.0292169 (PMC10624273; doi:10.1371/journal.pone.0292169)
Supplement: Attachment — Submitted filename: Response to Reviewers.docx [file pone.0292169.s002.docx]

**Factors leading to the late diagnosis and poor outcomes of breast cancer in Matabeleland South and Bulawayo Metropolitan Provinces in Zimbabwe**

*Magara MS et al*

**Rsponses to Editor’s Comments**

1. Manuscript style. *Response:* manuscript has been prepared according to PLOS ONE’s guidelines
2. Include full names of ethics committees that approved the study. *Response:* Medical Research Council of Zimbabwe, and National University of Science and Technology Institution Research Board (Lines 276- 278)
3. Ethics statement and consent. *Response:* see Lines 279-286)
4. In your Methods section, please provide additional information about the participant recruitment method and the demographic details of your participants. Please ensure you have provided sufficient details to replicate the analyses such as: a) a detailed description of any inclusion/exclusion criteria that were applied to participant recruitment, c) b) a description of how participants were recruited." *Response:* A range of criteria for inclusion encompassed respondents and organisations with community influence, a knowledge of BC, and women, and their relatives. Selected participants had acquired Ordinary Level qualifications, the minimum level of education that permitted comprehension of the NP objectives. People with no formal education were excluded. Participants were selected consecutively as they arrived at the hospitals. (Lines 323-328)
5. To comply with PLOS ONE submission guidelines, in your Methods section, please provide additional information regarding your statistical analyses. For more information on PLOS ONE' expectations for statistical reporting, please see <https://journals.plos.org/plosone/s/submission-guidelines.#loc-statistical-reporting>." *Response:* Quantitative data collected from patients and their relatives, and from HPs were aggregated from outreach areas in the MS Province districts as well as Bulawayo Metropolitan; and an SPSS Version 23™ package used for the analysis. Summary statistics like mean and mode were calculated for quantitative data and tests for associations using the chi-square test were carried out. Qualitative data from HPs, medical specialists, faith healers, hospital administrators, medical insurance companies, advocacy groups, charities and others were transcribed and analysed by NVIVO 9™ application (see Lines 437-450)
6. Discussion of limitations. *Response:* see Discussion under subtitle ‘Strengths and limitations of the study’, Lines 1191-1206)
7. We note that Figure 1 in your submission contains [map/satellite] images which may be copyrighted. *Response:* Figure 1 has now been omitted from the revised paper.
8. Besides, please supplement the manuscript with reference on the Cochran's Sample Size Formula. *Response*: Literature reference for the Cochran Sample size formulae was included. This is reference [15].
9. Show or describe the raw data belonging to Table 5 (now only the statistical test output is presented, without the underlying data). *Response*: The statistical analysis in **Table 2** was based on raw data in Annex table (iii).

| **Reviewer 1 comments** | **Authors’ response** |
| --- | --- |
| ***(1) Abstract*** Methods: the authors should clearly define the outcome variable, the method of data analysis, the study design | The dependent variable was cancer awareness among people in different districts and length of time taken by cancer patients to seek medical attention. Associations between quantitative variables were analysed using chi-squared test and qualitative data was analysed using frequency of similar statements or repetitions of similar statements from different districts and individuals. Most of the abstract has been re-written in response to these comments (Lines 139-152) |
| ***(2) Background*** The background section would be more interesting if the authors could expand the section to include more information about the available breast cancer diagnostics and treatment centers in the study hospital and better to compare it with other developing countries in Africa. | The Introduction section has been rewritten to provide a referenced up-to-date comparison with other countries in the region (Lines 171-178), Lines 204-209), and now describes the lack of availability of breast cancer facilities in municipal clinics, rural clinics and rural hospitals that do not have diagnostics centres or any resident doctors, who only visit according to predetermined rotas (Lines 185-198). These are the District Clinics which were the focus of the study. |
| ***(3) Methods*** Study area: it will be good if the authors should add more information about the study area (the availability of the diagnostic and treatment centers in the hospital, average patient flow per a specific period of time, the presence or absence of chemotherapy, radiotherapy services , available professionals on the area …I suggest the authors to clarify /to show the following points  *(i) Study populations and sample populations?* | The location, population demographics and provision of healthcare in the Needs Project study areas of Matabeleland South and Bulawayo Metroplitan Provinces is described on Lines 250-271), with contemporaneous references  (i) The study popualtions included residents from 6 districts in Matabeleland South (Lines 320-323), and from 2 Bulawayo City Clinics. The sample populations are described in Lines 329-354) |
| *(ii) Inclusion and exclusion criteria?* | (ii) The initial selection criteria were based on respondents and organisations with community influence, knowledge of BC, BC patients, and relatives. Participants were selected as they arrived at the hospitals, and were exluded if they had no formal education (Lines 323-328). |
| *(iii) Where was the ethical clearance was obtained?* | (iii)The NP was carried out in three phases, after Ethical Approval was granted by the National University of Science and Technology Institute Research Board (NUST IRB) Medical Research Council of Zimbabwe (Approval code MRCZ/A/2582 (Lines 276-278) |
| *(iv) How the participants are selected or included in the study?* | (iv) See response 4 above (Lines 323-328) |
| *(v) What was the sampling technique?* | (v) Sampling of participants from patient and relative groups and health professionals were quantitative and employed Cochran Sample Size formulae.  The Cochran Sample Size Formulae technique was used to determine the sample size of patients and their relatives since the population was large, that is 1 300 000. The sample was calculated to be 1100 respondents.  Bulawayo Metropolitan and Matabeleland South Provinces have an estimated population of 3500 health professionals. The Cochran Sample Size Formulae technique determined a sample size of 400 respondents.  Qualitative study participants from advocacy groups and health stake holders were selected through purposive and snowballing sampling techniques (Lines 329-354) |
| (*v) On the data analysis section, the authors should briefed about the statistical analysis in detail for quantitative data (descriptive statistics and inferential statistics) and the methods of qualitative data analysis (thematic content or* *framework analysis* | Summary statistics like mean and mode were calculated for quantitative data and test for associations using the chi-square test were done (Lines 437-449) |
| *(vi) Who transcribed the qualitative data?* | (vi) The research fellows / core investigators transcribed qualitative video and audio data to Microsoft Word™ documents (Lines 423-426) |
| *(vii) How the trustworthiness of qualitative data was assured (credibility, dependability, transferability, confirmability?* | (vii) The accuracy of the transcripts was verified with groups who had attended the focus group discussions before they were submitted for qualitative data analysis by the statisticians. Improved quality of data was assured through the training data collectors and multiple perspectives of data collection. In addition, theoretical triangulation, participant validation and member checks were conducted to ensure quality data was collected. These processes were managed by the principal investigator and core investigators (Lines 423-426) and (Lines 444-449) |
| *(viii) It will be good to mention the qualification and experience of those who conducted the qualitative data collection and analysis* | (viii) Qualitative data was collected by the Principal Investigator and Core Investigators with masters’ qualifications and records of qualitative research interests. A statistician who holds a master’s degree conducted the qualitative data analysis (Lines 444-446 and 449-450) |
| *(ix) Could you please justify the need of qualitative data since the objectives of the study can be addressed in qualitative methods alone?* | (ix) Qualitative data was collected from health professionals, medical specialists, faith healers, medical insurance companies, advocacy groups, charities and others using a combination of open-ended and closed questions by two surgeons and two research fellows / core investigators. Some objectives of the NP, for example the need to establish the sometimes complex reasons for a delayed BC diagnosis, are best answered through a dialogue exploring lived experiences, hence the need for qualitative data. Qualitative data also substantiated what quantitative data revealed (Lines 413-417; 446-449) |
| *(x) Framework: the authors present a conceptual framework, but there is no explanation for how it was developed and/or reference to any existing cancer early diagnosis framework developed by WHO or other public health agency. Furthermore, the presented framework is not used to help* *organize the content, leaving the reader without a clear reference to process the material presented. Using an existing framework can further orient the findings around key outcomes (beyond the target on late stage disease) such as delays in specific intervals along diagnostic pathway, treatment access and/or abandonment* | (x) The conceptual framework section has been deleted altogether. |
| ***(4) Results*** *(i) The tables can be merged and please minimize the number of tables (merge table 1.2.3.4 ) : the tables are not attractive since you prepare a table for a single variable*    (*ii) The result section is not informative and needs rewriting!! please look other papers conducted on late diagnosis of breast cancer in Africa*  *(ii) The figures are also need modification, please look journal guideline*  *(iii) For the qualitative data , please show the themes, categories and codes identified from the participant narrations*  ***(5) Discussion and conclusion*** (i) The discussion section needs comparing your results with other studies conducted globally and you have justify the similarities and difference between the studies; nothing you do in this area  (ii) What are the strengths and limitation of your study? | (i) Tables 1, 2 ,3 and 4 merged, see Table 1 (Lines 474-477)  Table 5 is now Table 2  Table 9 replaced by Table 3  Tables 12,13 and 14 merged, see Table 4  Tables 16 and 17 merged, see Table 5  Table 6 now replaces and summarises the entire section describing the responses to the qualitative questionnaires (Lines 863-866)    (ii) The results section has been almost completely rewritten, all Tables have been extensively modified (as above), and several of the original Tables and Figures have been referred to in the text, and moved to the Annex (Lines 451-866). A number of contemporaneous papers on the same topic have been included and compared in the Discussion section (Lines 1159-1174)  (ii) Format of Figures corrected.  (iii) See ‘issues and key messages’, Table 6 (Lines 863-866), and ‘key messages’ subsection in Discussion section (Lines 1112-1156)  (i) Comparisons with Global and other Afrcan studies have been made in the Introduction (Lines 169-178) and in the Discussion section (1159-1174)  (ii) This aspect has been addressed in the Discussion section (Lines 1191-1206) |
| **Reviewer 2 Comments** |  |
| **A. Major considerations** ***1. Presentation of findings and length****: there are many significant findings that are informative. However, a significant proportion of the results can be moved to an Annex allowing for more streamlined, efficient narrative that highlights key points.*  *Emphasis should be made on the results that support the primary conclusions.*  *For example, (a) Knowledge of breast cancer by district and/or work experience by District: what implications do these data have? Can this be deleted? If included, is there are association between the two?*  *(b)Demographics and characteristics of respondents and health professionals can each be summarized in one table (and cross tabulation of age and educational levels can be presented without a tabl)e*  . *(c) Data can also be better presented to improve the efficiency of the content. For example, the FGDs can be consolidated into one major section, focusing on key findings and any relevant differences identified.*  *(d) Content like “Word Clouds” can also be moved to an Annex.* | (i) The paper has undergone a major revision. The length of the text has been reduced by 33% from ~8,700 to ~5,800 words.  The number of tables has been reduced from 19 to 6 by a combination of (i) merging some (eg parts of Tables 1, 2 ,3 and 4 merged, see Table 1 (Lines 474-477) and (ii) relocating others to the Annex (eg parts of (original) Tables 1+4, and Tables 6,7,10,11,14-18).  The findings of the qualitative study have been condensed into a single table (Table 6, text previously spanning Pages 42-50), and the ‘Word Cloud’ (original Figure 6) has been moved to the Annex. The findings of the study have been grouped and discussed under 7 ‘Key messages’ (Lines 1112-1156).  (a) The original Table 16 has been moved to the Annex, with a brief explanation of the findings of the quantitative study in the Results (lines 752-757), and of the qualitative study in Table 6 under ‘Awareness’. An analysis and proposed interventions are discussed under the first ‘Key message’ (Lines 1112-1156), and under “Interventions with potential to drive change’ (Lines 1209-1234)  (b)Tables 1, 2 ,3 and 4 have been merged, see Table 1 (Lines 474-477)  (c) All data collected from the FDGs has now been condensed into 1 short section, and summarised in a Table 6 (Lines 863-866)  (d) The ‘Word Cloud’ has been moved to the Annex. |
| 2***. Framework:*** *the authors present a conceptual framework, but there is no explanation for how it was developed and/or reference to any existing cancer early diagnosis framework developed by WHO or other public health agency. Furthermore, the presented framework is not used to help organize the content, leaving the reader without a clear reference to process the material presented. Using an existing framework can further orient the findings around key outcomes (beyond the target on late stage disease) such as delays in specific intervals along diagnostic pathway, treatment access and/or abandonment.* | The conceptual framework section has been deleted altogether. |
| ***3. Statistical review:*** *there are several additional analyses that could improve the data quality. For example, (a) Sub-groups: multiple times in the study, a sub-group is presented without clarification as to why the provided data do not sum to 100% or to the group percentage (eg, lines 345-346, 349-350, 356-357)*  (*b) Analyses do not consider confounding factors. For example, the awareness of cancer incidence should be done by age and by profession.*  *(c) Additionally, clarification should also be provided regarding select results/findings. For example, what is the difference between “No” and “Don’t Know” as it pertains to “Knowledge about breast lumps (Table 6)?*  *(d) Also, how why is inadequate medicine considered a delay when not all breast cancers require medicines?* | (a) Regarding subgroups, the reasons for not adding up to 100% have been included in the document. A proportion of respondents did not give a response to each question.  (b) Awareness of BC by age group is summarised in Table 5 (Lines 740-742). The significant association between age and awareness, and the lack of an association between awareness and profession is highlighted (Lines 722-730)  (c) The text has been rewritten (Lines 528-534) to clarify this point.  Participants responding ‘No’ had no idea/did not know about breast lumps, and those reponding ‘Don’t know’ were not sure about their nature.  (d) ‘Delay’ refers to delay of conventional BC drug treatment (Lines 614-621), which is considered an essential part of disease management in >95% BC patients. The reasons for undertreatment are discussed (Lines 1113-1156), including a belief in traditional medicine in Zimbabwe |
| **B. Minor Considerations** |  |
| *(i) Key findings should be clarified and made more coherent: there are recommendations for greater specialization and also decentralization. How would the authors advise policy-makers to interpret these 2 findings in the short-term through a coherent approach?* | (i) The key findings are now summarised in the Discussion section, subsection ‘Key messages’, as a list of the distinct reasons leading to delay at every stage of a woman’s pathway from awareness to treatment of BC (Lines 1112-1156).  Our advice to policy-makers is laid out in the last section of the Discussion (Lines 1209-1234) |
| *(ii) Survey questions: the inclusion of “Have you ever discovered a breast lump” and “Did someone ever examine you and find a breast lump?” would be informative to help guide primary care planning. These data are not well known and were valuable to have included in the questionnaire. Furthermore, can further insights be provided on the women who travel abroad to seek care? This is also particularly relevant to understand.* | (ii) ‘Have you ever discovered a breast lump’- the question aimed to find out the number of women who discovered lumps by performing breast self-examination  ‘Did someone ever examine you and find a breast lump’- the aim was to find out the number of women who had breast lumps discovered in a formal clinical examination.  While it is known that women travel abroad for treatment of breast cancer, the exact numbers cannot be accounted for because most patients travel informally in their individual capacities. It is believed that a total of 17000 of Zimbabweans who travelled to South Africa in 2010 did so for medical reasons. That figure constituted 3% of all Zimbabwean travellers to RSA that year. (Crush, Chikanda and Maswikwa, 2012, Southern African Migration Programee (SAMP), 2012). Idasa, Cape Town). In view of the uncertainties about numbers travelling, and the reasons, these details have not been included in the text. |
| *(iii) VIAC voucher system: given that this is part of the recommendations, it would be helpful to provide the reader with additional background.* | (iii) See additional background and description of this system and implications for BC clinics in the Discussion section (Lines 1176-1190) |
| (*iv) MoH: was consideration given to interview Ministry of Health representatives as a key stakeholder? If not, why not?* | (iv) The Provincial Medical Officer of Matabeleland South Province (Ministry of Health and Child Care’s highest ranking medical officer in the province) was represented at the Stakeholders’ interviews, and has subsequently become a major partner for the next phase of the project. |
| *(v) Data collectors: how many were trained?* | (v) A total of 14 data collectors were trained. Each of the 6 districts had 2 data collectors, and Bulawayo City Clinics also had 2 data collectors (Lines 383-395) |
| *(vi) “Patients and relatives”: can an exact breakdown be provided?* | (vi) ‘Patients and relatives’ were treated as a single group. The exact breakdown was never made. |
| *(vii) Reference: please add reference for sentence 102-104 (and consider exchanging “manpower” with “health workforce”* | (vii) The National Cancer Prevention and Control Strategy for Zimbabwe 2013-2017. (Ministry of Health and Child Care, Harare, 2013) This is Reference 12, cited in lines 202 and 216) of the Introduction.  ‘Workforce’ now used in Line 205 in rewritten Introduction. |
| *(viii) Breast cancer as witchcraft: this was referenced in the FGD but not reflected to the same extent in the stakeholder interviews (eg, Table 8). Can the authors provide their interpretation of the frequency of such belief systems in the Discussion?* | (viii) Patients and relatives living in rural settings believe that BC is due to their traditional strong belief in witchcraft, more frequently than those living in urban settings. Here, better education leads to a better understanding of the medical aspects of BC, and reduces their belief in witchcraft.. This is referred to in the Discussion under ‘lack of trust’ (Lines 1127-1131), and in Table 6, section ‘Treatment’, (Lines 863-866) |
