# Supplementary material for: Factors leading to the late diagnosis and poor outcomes of breast cancer in Matabeleland South and the Bulawayo Metropolitan Provinces in Zimbabwe
Source: PLoS One. 2023 Nov 3;18(11):e0292169. doi: 10.1371/journal.pone.0292169 (PMC10624273; doi:10.1371/journal.pone.0292169)
Supplement: Attachment — Submitted filename: Response to Reviewers Comments.docx [file pone.0292169.s003.docx]

**Response to Reviewers’ Comments**

1. If the authors have adequately addressed your comments raised in a previous round of review and you feel that this manuscript is now acceptable for publication, you may indicate that here to bypass the “Comments to the Author” section, enter your conflict of interest statement in the “Confidential to Editor” section, and submit your "Accept" recommendation.

Reviewer #1: All comments have been addressed

Reviewer #3: (No Response)

2. Is the manuscript technically sound, and do the data support the conclusions?

The manuscript must describe a technically sound piece of scientific research with data that supports the conclusions. Experiments must have been conducted rigorously, with appropriate controls, replication, and sample sizes. The conclusions must be drawn appropriately based on the data presented.

Reviewer #1: Yes

Reviewer #3: Partly

3. Has the statistical analysis been performed appropriately and rigorously?

Reviewer #1: Yes

Reviewer #3: Yes

4. Have the authors made all data underlying the findings in their manuscript fully available?

Reviewer #1: Yes

Reviewer #3: Yes

5. Is the manuscript presented in an intelligible fashion and written in standard English?

PLOS ONE does not copyedit accepted manuscripts, so the language in submitted articles must be clear, correct, and unambiguous. Any typographical or grammatical errors should be corrected at revision, so please note any specific errors here.

Reviewer #1: Yes

Reviewer #3: No

6. Review Comments to the Author

Please use the space provided to explain your answers to the questions above. You may also include additional comments for the author, including concerns about dual publication, research ethics, or publication ethics. (Please upload your review as an attachment if it exceeds 20,000 characters)

Reviewer #1: The manuscript is potential for publications . The authors addressed my comments in the revised document

Reviewer #3:

1. The framing of sentences in the manuscript is poor which needs to be rephrased.

*It's difficult to address this comment without knowing which sentences/paragraphs your Reviewer is referring to, short of rephrasing/rewriting the whole document. This issue was not raised in relation to either of the 2 previous submissions, or indeed by any of your previous reviewers. The senior author carried out all final edits, including this third iteration, and has published extensively without encountering any comments of this kind*. *We trust that any concerns of this nature have now been addressed satisfactorily in the sections below that have been revised in line with Reviewer 3’s suggestions.*
2. The materials and methods must descriptive at the same time it should be on point and precise. For example- The population details of MS and Bulawayo Metropolitan provinces is not necessary in the methodology section.

*The population details have been moved to the opening paragraph of the discussion (Lines 581-586).*
3. Statement regarding ethical approval has to come in the end section of materials and methodologies.

*This statement has been moved to the end of the materials and methods section under a new subtitle ‘Ethical approval and consent’ (Lines 288-300)*
4. The various steps conducted in the study methodology process can be written in precise manner, highlighting the important points.

*See point 5 below.*
5. The sub-headings and contents of the materials and methods section needs reframing. For example- Justification for Methodology can be written as Research Design and Qualitative data section has been poorly framed.

*The subtitle ‘Justification for Methodology’ has been replaced by ‘Research Design’ (Line 303), and the ‘Qualitative data’ subsection has been rewritten to clarify the rationale and methods used during the qualitative data collection phase (Lines 317-333).*
6. The legends of the tables also needs to be re-consider. For example- Table 1- Demographic characteristics of the respondents.

*Legends of Tables 1, 3 and 4 changed* *as suggested.*
7. Table 2 is not giving a clear description of the significant association between the variables used for Breast Cancer awareness. Hence, restructuring of the table need to be done incorporating the factors used for the significant test.

*Table 2 and associated text have been revised as suggested, now incorporating the parameters for tests of association. (Lines 404 – 414).*
8. Discussion part has been poorly framed.
First part of discussion should discuss the brief details of the study and its prime findings.

*See new subsection of Discussion ‘Summary of the study and findings’ (Lines 579-602).*
Second part of discussion should compare the findings with the international studies done under similar objectives with rationale.

*See new subsection of Discussion ‘Comparison with international studies’, with 6 additional references (Lines 604-619).*
Third part of discussion should compare the findings with the national studies done under similar objectives with rationale

*See new subsection of Discussion ‘Comparison with national studies’, with one additional reference (Lines 623-646).*
Fourth part of discussion should be the superficial inference drawn for this study citing the strengths.

*See new subsection of Discussion ‘Strengths of the study’ (Lines 648-674)*
9. Key messages mentioned in this manuscript should be after discussion part.

*74See new position of ‘Key messages’ (Lines 680-774).*
10. The sequence should be-
1. Discussion

*See relocated ‘Discussion’ section (Lines 578—674).*
2. Key messages

*See new position of ‘Key messages’ (Lines 680-731).*
3. "Advice to policy- makers implementing changes" (Change it to - Potential strategies for policy-makers)

*See new position ‘Potential strategies for policy-makers’ (Lines 776-801).*
4. Limitations of the study

*See new position for ‘Limitations of study’ (Lines 803-848).*
5. Conclusions

*See new position for ‘Conclusions’ (Lines 877-889).*
6. Acknowledgements

*See new position for ‘Acknowledgements’ (Lines 896-899).*
7. References

*See new position for ‘References’, with seven additional references relating to (Lines 901-1001)*
